# Association of Anxiety/Depressive Symptoms with Psychotic-like Experiences: The Moderation Effect of Sex and Resilience

**DOI:** 10.3390/children11080969

**Published:** 2024-08-11

**Authors:** Manling Long, Peiyu Zhang, Jingyu Shi

**Affiliations:** 1 School of Medicine, Tongji University, Shanghai 200331, China; 2311053@tongji.edu.cn (M.L.); zhangpy@tongji.edu.cn (P.Z.); 2 Clinical Research Center for Mental Disorders, Shanghai Pudong New Area Mental Health Center, Tongji University School of Medicine, Shanghai 200124, China; 3 Department of Medical Humanities and Behavioral Sciences, School of Public Health, Tongji University School of Medicine, Shanghai 200331, China

**Keywords:** psychotic-like experiences, adolescents, sex, resilience, depression, anxiety

## Abstract

Background: Psychotic-like experiences (PLEs) are a part of the continuum of psychosis and are common in the general population. While most of these experiences are transient, they are strongly correlated with an increased risk of various adverse psychological outcomes. Anxiety and depressive symptoms also occur frequently in the adolescent population. Much research has previously demonstrated a correlation between these two symptoms and PLEs. However, few investigations have examined what influences this association, and sex and resilience may be important moderators. Methods: This study selected a sample of first-year students from a university in Shanghai. A total of 2970 adolescents completed questionnaires measuring sociodemographic characteristics, anxiety/depressive symptoms by SCL-90, resilience by CD-RISC, and self-reported PLEs by PQ-16. Results: The findings indicated that PLEs were prevalent in the sample, with at least one PQ-16 item present in 42.5% of individuals. Anxiety/depressive symptoms were significantly associated with PLEs, and there was a sex difference in this association (*p* < 0.001). What is more, this relationship was stronger in males than in females. Additionally, we found a significant interaction (*p* < 0.001) between resilience and anxiety/depressive symptoms when looking at the correlates of PLEs. Those with stronger resilience showed a considerably weaker connection between PLEs and symptoms of anxiety and depression. Conclusions: These findings can potentially inform the development of targeted new clinical interventions.

## 1. Introduction

Psychosis is a continuum, which means that the same symptoms as those in people with psychotic disorders can also be observed in the general population, and we refer to this subthreshold psychotic symptomatology that occurs in the general population and does not reach a clinical diagnosis as a psychotic-like experience (PLEs), which has been found to have a prevalence of approximately 7–27% in the general population in previous studies [1,2]. Although transient for most people, PLEs can be distressing [3], and a large body of research suggests that they may lead to psychotic [4,5] and non-psychotic disorders [6], as well as increased risk for a variety of adverse outcomes [7,8,9].

The possible link between risk factors for schizophrenia and PLEs has been widely reported in previous studies. Various factors such as genetic characteristics, family history of mental illness, adverse childhood experiences, unfavorable social conditions (e.g., low socioeconomic status, unemployment, marital breakup, etc.), marijuana abuse, and various prenatal and perinatal complications are strongly associated with the development of PLEs [10,11,12,13,14]. Anxiety and depressive symptoms have received much attention in the context of psychiatry and have been the focus of a large number of studies. Both symptoms are highly general in individuals with psychosis [15] and those at ultra-high risk for psychosis [16]. Varghese and colleagues [17] have shown that persons with major depression or anxiety disorders are at a fourfold increased risk of delusions. Moreover, anxiety and depression can exacerbate psychotic symptoms, increase discomfort in patients, harm the course of treatment [18], and may also increase the likelihood of disease recurrence and make treatment more difficult [18]. Increased chances of experiencing PLEs can be associated with anxiety/depressive symptoms interacting with environmental factors [19]. Anxiety, depression, and PLEs might be construed as different dimensions of a continuous spectrum by certain scholars [20], with PLEs falling at the more extreme end of this spectrum. Several pieces of evidence support the firmly established connection between anxiety/depressive symptoms with PLEs. It has been indicated [21,22,23] that anxiety and depressive symptoms showed significant associations with PLEs and often served as mediating variables linking risk factors to PLEs. Further, in observations from longitudinal studies, we have identified a similar pattern of change between PLEs and anxiety/depressive symptoms, with overlap between the two [24,25]. However, despite some confirmation of this association, little research has been conducted on the variables that could affect this association. Future research is expected to be carried out to determine the primary determinants of this connection.

Sex differences have been a very important part of the research on schizophrenia and related disorders [26,27]. The studies about PLEs do not lack a comprehensive examination of sex variables as well. Although the issue of sex differences in PLEs is not yet settled, much research has explored it, presenting a variety of perspectives and findings. Some studies have indicated that females had a higher prevalence of PLEs [28,29], while others have found that males had a higher prevalence [30,31]. However, these differences were not found in a large sample study of Chinese adolescents [32]. Much attention has also been focused on sex differences in anxiety and depression. One meta-analysis [27] has indicated that depressive symptoms exhibited a significant sex difference, with this difference typically reaching its peak around the age of 16. However, some studies have not identified such sex differences in anxiety or depressive symptoms in the general population [33]. Nevertheless, further research is required to ascertain whether sex interacts with anxiety/depressive symptoms and has an impact on PLEs.

Resilience, as an important protective factor for mental health, refers to the process by which an individual demonstrates a well-adapted capacity in the face of stressful situations such as adversity, grief, trauma, and threat [34]. The presence of resilience allows an individual to recover quickly from difficult situations. Resilience also plays an important role in dealing with stress, solving problems, and maintaining a high quality of life and well-being [35,36,37]. According to risk protection theory [38], resilience, through its interaction with risk factors, can effectively reduce the potential risk to an individual’s mental health and, thus, play an important protective role. For instance, there is clear evidence demonstrating the positive role of resilience in moderating the relationship between sleep disorders and PLEs in adolescents [39], and similar protective effects have been found between depressive/anxiety symptoms and suicidal ideation [40]. It was also pointed out in several previous studies that resilience was one of the important protective factors for anxiety/depressive symptoms and PLEs [41,42,43]. At present, however, we are still unclear whether resilience is effective in buffering the potential impact of anxiety/depressive symptoms on PLEs. We hope that further studies will be conducted to clarify further mechanisms underlying the role of resilience in mental health.

Our study wanted to examine PLEs and their correlates in this sample of adolescents. We further discussed sex differences between anxiety/depressive symptoms and PLEs, as well as whether resilience could play a buffering role in the impact of anxiety/depressive symptoms on PLEs. Our hypotheses are as follows:

i Anxiety and depressive symptoms were found to have a positive association with PLEs, while resilience showed a negative association with PLEs;
ii There were sex differences in the association, which were more significant in males compared to females between anxiety/depressive symptoms and PLEs;
iii Individuals with lower levels of resilience exhibited stronger associations between anxiety/depressive symptoms and PLEs in comparison to individuals with higher levels of resilience.

## 2. Materials and Methods

### 2.1. Study Participants

The cross-sectional survey, which included all freshmen in this study, was conducted fully at a comprehensive university in Shanghai. A total of 3021 questionnaires were distributed for the research, and 2970 of these were returned, resulting in a response rate of approximately 98.3%. After fully understanding the purpose and content of this study, all participants signed a written informed consent form. Written consent was also obtained from the parents or guardians of participants under the age of 18. The completion of the questionnaire was supervised by the researcher and took approximately 15–20 min. This study was reviewed and formally approved by an ethics committee, and all participants were informed of their right to voluntarily participate or withdraw from this study at any time.

### 2.2. Measurements

#### 2.2.1. Psychotic-like Experiences (PLEs)

The 16-item version of the Prodromal Questionnaire (PQ-16) was designed as a self-report questionnaire to screen for the presence of PLEs and the distress of PLEs [44]. The questionnaire assesses the presence or absence of PLEs using a rating scale from 0 (no) to 1 (yes), and the total score ranges from 0 to 16. In addition, the PQ-16 questionnaire also evaluated the level of distress in PLEs, with scores ranging from 0 (no distress) to 3 (severe distress) and total scores ranging from 0 to 48. A previous study has already confirmed the reliability and validity of the Chinese version of this questionnaire [45]. In the present study, we paid particular attention to distress scores as a continuous variable in the PQ-16. This way enabled us to explore the effects of PLE and related distress more thoroughly. The Cronbach alpha value for the distress score in this study was 0.84.

#### 2.2.2. Resilience

The Chinese version of the Connor–Davidson Resilience Scale (CD-RISC) [46] was used to assess an individual’s level of resilience in this study. This scale consists of three core dimensions: resilience; strength; and optimism. There are 25 items, each of which is rated on a five-point Likert scale, with specific scores ranging from 0 (never) to 4 (always). The total score on the scale ranges from 0 to 100. The higher scores indicate higher levels of individual resilience. In this study, the reliability of the CD-RISC was assessed by Cronbach’s alpha coefficient, which was 0.94, indicating good internal consistency.

#### 2.2.3. Depression and Anxiety

Anxiety and depressive symptoms were assessed using two separate subscales of the Chinese version of the Symptom Check-List-90(SCL-90) [47]. The depression subscale and the anxiety subscale were employed to evaluate whether participants exhibited symptoms of depression or anxiety in the previous two weeks, respectively, and to quantify the severity of these symptoms. The anxiety and depression subscales contain 10 and 13 specific items, respectively. Each item is rated on a five-point Likert scale, with a scale of 1 to 5 corresponding to five different levels of description: none; very mild; moderate; severe; and most severe. Subsequently, depression scores (ranging from 13 to 65) and anxiety scores (ranging from 10 to 50) were derived by summing the scores of the entries belonging to each subscale. The higher the scores, the more severe the individual’s anxiety or depression.

#### 2.2.4. Other Variables

In addition to the variables mentioned above, we examined sociodemographic variables that were closely associated with PLEs. These included gender, age, leave-behind experience (yes or no), presence of a family history of mental illness (yes or no), as well as family relationships and monthly household income. To assess family relationships, a four-point Likert question was employed, with scores increasing from 1 (indicating very poor) to 4 (indicating good). In addition, the monthly household income was divided into five categories, specifically as follows: below RMB 2000; RMB 2000–4999; RMB 5000–9999; RMB 10,000–19,999; and RMB 20,000 and above.

### 2.3. Statistics

In this study, IBM SPSS 25.0 software was used to analyze the main variables with comprehensive and detailed descriptive statistics. To deeply analyze the differences between categorical and continuous variables among adolescents of different sexes, one-way ANOVA (including chi-square test and independent samples *t*-test) was used. Meanwhile, by calculating Pearson’s correlation coefficient between the main variables, we further revealed the intrinsic relationship between them. On this basis, we further focused on the sex differences in the relationship between anxiety and depressive symptoms and PLEs and explored the role of resilience in this process. After standardizing all continuous variables, taking full account of the effects of multiple covariates, such as age, family history of mental illness, family relationships, and family experiences, we used the PROCESS software macro for SPSS (Model 1) to test the moderating effects of sex and resilience, testing by bootstrapping using the Bootstrapping (n = 5000) and 95% confidence intervals to assess indirect effects. The results were considered statistically significant if the 95% CI did not include zero and the *p*-value was less than 0.05. Finally, for variables with significant interactions, we performed further regression analyses or simple slope tests to verify and refine our findings.

## 3. Results

### 3.1. Sample Characteristics

The demographic characteristics of the sample and descriptive statistics for the variables are presented in Table 1. Through the results of statistical analysis, we found statistical differences between males and females on several variables. Specifically, males and females exhibited significant age differences (*p* < 0.01), family relationships (*p* < 0.05), household incomes (*p* < 0.001), self-reported PQ-16 scores (*p* = 0.001), and PQ-16 distress scores (*p* < 0.05). Males scored higher on PQ-16 distress. However, there were no significant differences in the left-behind experiences, family history of mental illness, depressive symptoms, anxiety symptoms, and resilience.

### 3.2. Correlations

The results of the correlational analyses (Table 2) suggested that males or those who reported a family history of mental illness had higher levels of PQ-16 distress scores. The higher levels of anxiety (r = 0.589, *p* < 0.001)/depressive symptoms (r = 0.568, *p* < 0.001), poorer family relationships (r = 0.158, *p* < 0.001), and lower levels of resilience (r = −0.310, *p* < 0.001) were the higher levels of PQ-16 distress. However, there were no significant relationships between age, left-behind experiences, and PQ-16 distress scores.

### 3.3. Sex Differences in the Relevance of Anxiety/Depressive Symptoms with PLEs

Following the adjustment of potential confounding variables, including age, family history of mental illness, family relationships, and left-behind experiences, we proceeded to examine the gender differences between depressive/anxiety symptoms and PQ-16 distress. Findings revealed a significant interaction between sex and depressive symptoms (β = 0.059, *p* < 0.001)/anxiety symptoms (β = 0.088, *p* < 0.001). To further clarify this relationship, we conducted linear regression analyses of anxiety/depressive symptoms and PQ-16 distress scores for each sex group (Table 3).

### 3.4. Association of Anxiety/Depressive Symptoms with PLEs: The Role of Resilience

**Figure 1:**
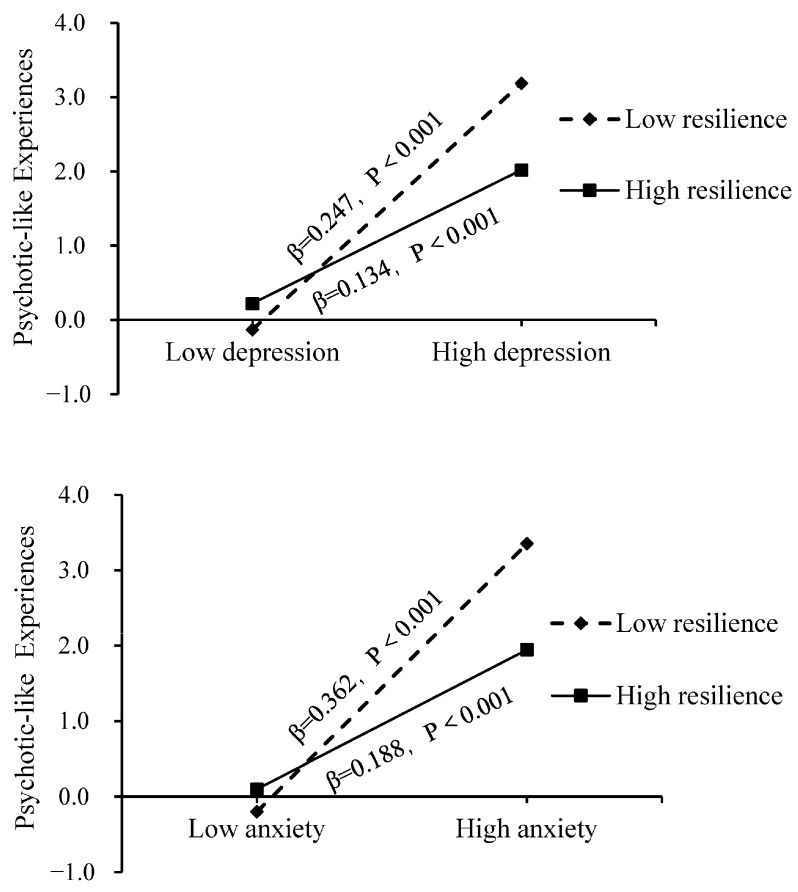
Plot of simple slopes for the interaction between depression/anxiety and resilience on PLEs.

We conducted a discussion on the interaction between resilience and symptoms of depression (β = −0.004, *p* < 0.001) and anxiety (β = −0.006, *p* < 0.001) behind controlling the aforementioned covariates. Moreover, a simple slope analysis has been performed to gain a more intuitive understanding of this interaction (Figure 1). The outcome suggested that individuals who had lower levels of resilience exhibited a stronger association between anxiety/depressive symptoms and PLEs. Conversely, individuals with higher levels of resilience demonstrated weaker associations in the relationship between anxiety/depressive symptoms and PLEs.

## 4. Discussion

The present study briefly investigated PLEs in this sample population, resulting in 42.5% endorsing the presence of at least one PQ-16 item. This detection rate is comparable to that observed in a previous study of Chinese university students [48]. The distress scores on the PQ-16 demonstrated that males experienced significantly greater distress than females. The present study further examined the correlation between anxiety and depressive symptoms and PLEs. We found a sex difference in these relationships, which were between anxiety/depressive symptoms and PLEs, which were stronger observed in males than in females. Additionally, resilience was identified as a protective factor that mitigated the effects of anxiety/depressive symptoms on PLEs.

In the correlation analysis, we found many factors associated with PLEs. Our research revealed a significant correlation between family relationships [49], family history of psychiatric disorders [50], and PLEs, which have been proved in previous research. Additionally, we observed that PLEs were positively associated with anxiety and depressive symptoms [51,52,53]; rather, they exhibited negative associations with resilience. According to the affective pathway of psychosis [54,55], stress may influence PLEs through affective disorders. These combinations of mood dysregulation symptoms (anxiety/depressive symptoms) and environmental factors further increased the risk of PLEs [19,56,57]. Therefore, it is particularly important to examine the factors influencing the link between mood dysregulation symptoms and PLEs. Previous research has established that resilience played a protective role against a variety of adverse psychological outcomes. Specifically, resilience moderates adolescents’ mental health in the face of negative life events [58] and acts as a buffer in the association between sleep disturbance and PLEs [39]. Similar conclusions have been reached in our study, which further emphasized the importance of resilience as a protective factor against PLEs. Although previous studies have generally stressed the significance of age on PLEs [12,59] and noted that the probability of PLEs declines with age, no significant correlation between age and PLEs was observed in our study. This result may be related to factors such as our sample characteristics and research methodology. Further studies are needed to explore and validate this in depth.

Data analysis in the present study revealed that males and females did not show statistically significant differences in anxiety and depressive symptom scores; however, the mean scores of females were higher than males in anxiety symptoms, and these results are somewhat similar to those of a previous longitudinal study of Chinese university students [33]. Although a previous study [28] examined sex differences in the association between anxiety/depressive symptoms and distress in PLEs, few significant differences were found. However, the present study further dissected significant sex differences in the association between distress and anxiety/depressive symptoms induced by PLEs, specifically noting that this association was more pronounced in the male population. We think that these differences in findings may be due to a combination of factors, such as differences in scale selection, differences in participant screening criteria, and the effect of sample size. In addition, differences in the emotion regulation strategies that men and women use to manage emotions may also contribute to this sex difference [60]. Specifically, there are sex differences in emotion regulation strategies such as cognitive reappraisal (i.e., shifts in the way emotional events are understood) and expressive inhibition (i.e., self-regulation of emotional expression) [61], and these differences may lead to different manifestations at the level of psychopathology [62]. Previous research has generally concluded that women are more likely to express emotions directly [60], whereas men are more likely to suppress or avoid experiencing and expressing emotions. They may obtain higher scores in PLE assessments because of the tendency to suppress emotions [63]. However, females typically show more concern for their feelings and even those of others when compared to males [64,65]. Therefore, it is possible that this profound understanding of emotions could make them employ a wide range of ways to regulate these emotions which may not only help them establish a strong relationship with other people but also relieve them of stress resulting from PLEs.

After considering other factors, we concluded that there was always protection from resilience, which made the direct path between PLEs and depression as well as anxiety less strong than before. We used simple slope analysis and realized that as the level of resiliency increased, the association between anxiety/depressive symptoms and PLEs decreased gradually. The higher the resilience levels among teenagers, the higher their optimism and improved self-efficacy and problem-solving confidence [35,66]. The chances are high that these good characteristics will assist them in dealing with mood swings instigated by depression and anxiety that could help get rid of the painful feelings caused by PLEs. Anxiety/depression symptoms in adolescents could be termed as stressful events in the theoretical framework of the risk-protection model [38], while resilience is a modesty factor that mitigates the link between these stressful events (anxiety/depression) and negative psychological outcomes (PLEs). The results of this study further highlight the critical role of enhanced resilience training in relieving symptoms of anxiety and depression, as well as reducing PLEs. As well as promoting healthy attachment [67], resilience training has been shown to help people regulate their emotions better and affect the way their brains process emotions [68]. The significance of resilience with anxiety, depression symptoms, and PLE was further confirmed through results from a randomized controlled trial [38]. Further, results from the randomized controlled trial suggested that for college students experiencing mild PLEs with depressive symptoms, interventions that were focused on training on resilience could result in decreased levels of both anxiety as well as depression, whereas the distressing aspect of PLEs decreased. This further underscores the value of resilience in the mental health field.

The current research has several restrictions that mainly arise from the design of this study and the kind of assessment tools used. First, due to the cross-sectional study design, it was difficult for us to determine the temporal and causal relationships between anxiety/depressive symptoms and PLEs. In subsequent studies, we could investigate the causal relationship between anxiety/depressive symptoms and psychotic-like experiences using a prospective longitudinal study and cross-lagged modeling to explore the directionality between anxiety/depressive symptoms and psychotic-like experiences, in particular, whether there is a bi-directional relationship between the two and the role that sex and resilience play in this. On the other hand, a self-report scale was employed in the current study for assessing PLEs, probably yielding more false positives, hence potentially affecting [69]. However, even self-reported false-positive results may indicate potential adverse psychological consequences [70]. Another limitation of this study is that the presence or distress of PLEs cannot be considered a consistent predictor for ultra-high-risk syndromes or psychotic disorders [44]. Put differently, only by considering the PQ-16 questionnaire as the only tool, it is difficult for one to know if an individual has ultra-high-risk syndrome/psychotic condition. In addition, this study did not measure PLEs in the context of any more structured interviews or clinical diagnosis, thereby reducing the capability to extend findings to cohorts showing ultra-high-risk syndromes or psychological disorders with ultra-risk syndromes. Hence, in interpreting and utilizing the outcomes from this research, it is indispensable for these constraints to be taken into account. In future studies, we may be able to improve the validity of the scale by integrating the clinical interview with the self-report scale in more nuanced ways, such as frequency/duration of abnormal experiences and self-awareness.

## 5. Conclusions

The results of our search indicate that although there are many articles highlighting the strong association between anxiety, depression, and PLEs, few studies have explored in depth what factors moderate this association. Our study examined the factors moderating the relationship between anxiety/depression levels and PLEs and found that this relationship was stronger in males than females. This finding may be due to the fact that men express greater inhibition in emotion regulation, and it may be that targeting changes in the emotion regulation skills of male patients would be effective in the subsequent development of interventions. Resilience can lessen the strong connection between emotional stress and peculiar perceptual experiences (PLEs) by acting as a buffer. These results are crucial when it comes to evaluating or controlling PLEs. In particular, resilience training targeting adolescents with both anxiety/depressive symptoms and specific perceptual experiences may have a more pronounced effect, which requires further research and implementation.

## Figures and Tables

**Table 1:** Participants’ characteristics.

Variables	Total Sample	Males (N = 1816)	Females (N = 1154)	Statistic	*p*-Value
Age	18.575 (0.704)	18.604 (0.754)	18.529 (0.617)	t = −2.969	0.003 *
Family relationship	1.35 (0.711)	1.33 (0.676)	1.39 (0.763)	t = 2.223	0.025 *
Left-behind children					
yes	666 (22.4)	426 (23.5)	240 (20.8)	χ^2^ = 2.887	0.089
no	2304 (77.6)	1390 (76.5)	914 (79.2)		
Family history of mental illness					
yes	128 (4.3)	78 (4.3)	50 (4.3)	χ^2^ = 0.002	0.962
no	2842 (95.7)	1738 (95.7)	1104 (95.7)		
Monthly household income				χ^2^ = 78.600	<0.001 *
<2000	327 (11.0)	242 (13.3)	85 (7.4)		
2000–4999	902 (30.4)	622 (34.3)	280 (24.3)		
5000–9999	1029 (34.7)	575 (31.7)	454 (39.3)		
10,000–19,999	496 (16.7)	269 (14.8)	227 (19.7)		
≥20,000	216 (7.3)	108 (5.9)	108 (9.3)		
PQ-16 item				χ^2^ = 10.593	0.001 *
yes	1263 (42.5)	815 (44.9)	448 (38.8)		
no	1707 (57.5)	1001 (55.1)	706 (61.2)		
PQ-16 Distress	1.46 (2.860)	1.58 (3.074)	1.27 (2.473)	t = −3.110	0.002 *
Depression	19.52 (6.905)	19.52 (6.870)	19.51 (6.963)	t = −0.043	0.966
Anxiety	14.93 (4.899)	14.79 (4.868)	15.15 (4.942)	t = 1.954	0.051
Resilience	69.41 (14.509)	69.33 (14.613)	69.53 (14.350)	t = 0.357	0.721

* Statistically significant at a level of *p* ≤ 0.05. N = Number of participants.

**Table 2:** Descriptive statistics and correlations among major variables (N = 2970).

	1	2	3	4	5	6	7	8
1.PQ-16 Distress	−							
2. Age	0.020	−						
3. Sex	0.054 ***	0.052 ***	−					
4. Family relationships	0.158 ***	0.027 *	−0.042 **	−				
5. Left-behind children	−0.016	−0.024	0.031 **	0.013	−			
6. Family history of mental disorders	0.067 ***	0.003 ***	−0.001 ***	0.053 ***	0.017	−		
7. Depression	0.568 ***	0.019	0.000	0.211 ***	−0.190	0.081 ***	−	
8. Anxiety	0.589 ***	−0.005	−0.036 *	0.193 ***	−0.007	0.077 ***	0.817 ***	−
9. Resilience	−0.310 ***	−0.031	−0.005	−0.190 ***	0.004	−0.101 ***	−0.438 ***	−0.379 ***

* *p* ˂ 0.05, ** *p* ˂ 0.01, *** *p* ˂ 0.001. Sex: female(0),male(1); Left-behind children: Yes(1), No(0); Family history of mental disorders: Yes(1), No(0).

**Table 3:** Regression coefficients for the separate linear regressions for females and males.

	Variables	B	β	*p*-Value	95%CI
Females	Depression	0.198	0.560	<0.001	(0.181, 0.215)
	Anxiety	0.291	0.582	<0.001	(0.268, 0.315)
Males	Depression	0.259	0.579	<0.001	(0.243, 0.276)
	Anxiety	0.381	0.603	<0.001	(0.357, 0.404)

## Data Availability

The data presented in this study are available on request from the corresponding authors. The data are not publicly available due to ethical restriction.
